# Weighted Gene Co-expression Network Analysis Identifies Crucial Genes Mediating Progression of Carotid Plaque

**DOI:** 10.3389/fphys.2021.601952

**Published:** 2021-02-05

**Authors:** Mengyin Chen, Siliang Chen, Dan Yang, Jiawei Zhou, Bao Liu, Yuexin Chen, Wei Ye, Hui Zhang, Lei Ji, Yuehong Zheng

**Affiliations:** ^1^Department of Vascular Surgery, Peking Union Medical College Hospital, Chinese Academy of Medical Sciences and Peking Union Medical College, Beijing, China; ^2^Department of Computational Biology and Bioinformatics, Institute of Medicinal Plant Development, Chinese Academy of Medical Sciences and Peking Union Medical College, Beijing, China

**Keywords:** carotid plaque, weighted gene co-expression network analysis, gene expression omnibus, crucial genes, RNA sequencing

## Abstract

**Background:**

Surface rupture of carotid plaque can cause severe cerebrovascular disease, including transient ischemic attack and stroke. The aim of this study was to elucidate the molecular mechanism governing carotid plaque progression and to provide candidate treatment targets for carotid atherosclerosis.

**Methods:**

The microarray dataset GSE28829 and the RNA-seq dataset GSE104140, which contain advanced plaque and early plaque samples, were utilized in our analysis. Differentially expressed genes (DEGs) were screened using the “limma” R package. Gene modules for both early and advanced plaques were identified based on co-expression networks constructed by weighted gene co-expression network analysis (WGCNA). Gene Ontology (GO) and Kyoto Encyclopedia of Genes Genomes (KEGG) analyses were employed in each module. In addition, hub genes for each module were identified. Crucial genes were identified by molecular complex detection (MCODE) based on the DEG co-expression network and were validated by the GSE43292 dataset. Gene set enrichment analysis (GSEA) for crucial genes was performed. Sensitivity analysis was performed to evaluate the robustness of the networks that we constructed.

**Results:**

A total of 436 DEGs were screened, of which 335 were up-regulated and 81 were down-regulated. The pathways related to inflammation and immune response were determined to be concentrated in the black module of the advanced plaques. The hub gene of the black module was *ARHGAP18* (Rho GTPase activating protein 18). *NCF2* (neutrophil cytosolic factor 2), *IQGAP2* (IQ motif containing GTPase activating protein 2) and *CD86* (CD86 molecule) had the highest connectivity among the crucial genes. All crucial genes were validated successfully, and sensitivity analysis demonstrated that our results were reliable.

**Conclusion:**

To the best of our knowledge, this study is the first to combine DEGs and WGCNA to establish a DEG co-expression network in carotid plaques, and it proposes potential therapeutic targets for carotid atherosclerosis.

## Introduction

Carotid atherosclerosis is characterized by lipid accumulation and inflammation, which underlie the thickening of the carotid intima where the plaque is formed (Libby et al., 2011). Compared with early plaques, advanced plaques are more vulnerable and prone to rupture. Surface rupture of the plaque leads to abrupt thrombus formation which, in turn, triggers cerebrovascular disease, including transient ischemic attack and stroke (Golledge et al., 2000).

In recent years, the rapid progress of microarray and RNA-seq technologies has facilitated gene expression profiling. It has been determined that hemoglobin metabolism and bone resorption are crucial pathways in plaque vulnerability, and dysregulated genes, including *SYNPO2*, *LMOD1* and *PPBP*, have been identified in carotid plaques (Perisic et al., 2016). In addition, Alloza et al. (2017) reported from an RNA-seq-based transcriptomic study that smooth muscle cells (SMCs) from unstable plaques showed a senescence-like phenotype, while stable plaques were suggestive of an osteogenic phenotype. A large meta-analysis of GWAS implicated one novel locus (*PIK3CG*) involved in carotid plaque and eight novel susceptibility loci linked with carotid artery intima thickness (Franceschini et al., 2018). Despite this progress, the molecular mechanisms involved in the formation and progression of carotid plaques have not been fully elucidated.

Weighted gene co-expression network analysis (WGCNA) is a powerful tool to identify gene co-expression modules, explore the correlation of the modules and phenotypes and discover hub genes that regulate critical biological processes (Zhang and Horvath, 2005). WGCNA has been widely employed in the cardiovascular field to investigate such topics as abdominal aortic aneurysms, obstructive coronary artery disease and varicose veins (Liu et al., 2016; Chen et al., 2019; Mo et al., 2019; Wang Y. et al., 2019; Zhang et al., 2019).

To clarify the potential molecular mechanisms underlying carotid atherosclerosis, we analyzed 2 datasets of transcriptomes downloaded from the Gene Expression Omnibus (GEO)^1^ and Sequence Read Archive (SRA) databases. Differentially expressed gene screening was conducted, and co-expression networks were constructed by WGCNA. Gene Ontology (GO) and Kyoto Encyclopedia of Genes Genomes (KEGG) pathway analyses were conducted in each module, and hub genes in each functional module were identified. Next, the DEG co-expression network was created, and crucial genes were mined based on this network using Molecular Complex Detection (MCODE). These crucial genes were validated using another independent dataset, GSE43292, through the WGCNA pipeline. Gene set enrichment analysis for 3 crucial genes was performed, and the fraction of 22 immune cells was determined and compared between early and advanced plaques by CIBRTSORT (Newman et al., 2015). Least absolute shrinkage and selection operator (LASSO) regression and linear discriminant analysis (LDA) were also performed to build a classifier to discriminate between advanced plaque and early plaque. Finally, a sensitivity analysis was performed to evaluate the robustness of the network we constructed.

## Materials and Methods

### Datasets

Three datasets, GSE28829, GSE104140, and GSE43292, were selected from the GEO database. GSE28829 and GSE104140 were used for WGCNA, while GSE43292 was used as the validation dataset. GSE28829 and GSE43292 were mRNA microarray datasets. GSE28829 involved 16 advanced plaque samples and 13 early plaque samples, while GSE43292 involved 32 atheroma plaques and paired macroscopically intact tissue. The series matrix file and data table of the microarray platforms GPL570 and GPL6244 were downloaded. GSE104140 was an RNA sequencing dataset that involved 19 advanced plaque samples and 13 early plaque samples. The fastq RNA sequencing data were downloaded from the SRA database (SRP118628). This study involved no human or animal subjects.

### Data Preprocessing and DEG Screening

Data tables of GPL570 and GPL6244 were used to annotate the series matrix files of GSE28829 and GSE43292, respectively, with official gene symbols (i.e., replace the probe name with the official gene symbol), and the gene expression matrices were obtained. Spliced Transcripts Alignment to a Reference (STAR) software (Dobin et al., 2013) was used to conduct RNA quantification to obtain the expression matrices of GSE104140. Next, we merged the gene expression matrix of GSE28829 and the TPM matrix of GSE104140. The “sva” R package was employed to remove batch effects (Supplementary Figure S1). Finally, the “limma” R package was utilized to conduct differentially expressed gene (DEG) screening. | log_2_ (fold-change)| >1 and adjusted *p* < 0.05 were set as thresholds of DEG screening. DEG analysis was also conducted for the validation set GSE43292, and we compared all the DEGs (adjusted *p* < 0.05) between the training set (GSE28829 and GSE104140) and this validation set.

### Co-expression Network Construction by WGCNA

The “WGCNA” R package was employed to construct co-expression network for both advanced plaque samples and early plaque samples. All the genes were involved in further analysis. Pearson’s correlation matrix was calculated. Next, using the formula a_mn_ = | c_mn_| ^β^ (where a_mn_ represents adjacency between gene m and gene n, c_mn_ represents Pearson’s correlation coefficient between gene m and gene n, and β represents soft-power threshold), the weighted adjacency matrix was created. A topological overlap measure (TOM) was created based on an adjacency matrix for gene module detection. In the detection of gene modules, average linkage hierarchical clustering was employed to build a clustering dendrogram, and the minimal gene module size was 100. After gene module detection, similar modules were merged with the threshold of 0.25. The atheroma samples in the validation set (GSE43292) were also included in another WGCNA.

### Functional and Pathway Enrichment Analysis

The “clusterProfiler” R package was employed for gene ontology (GO) and Kyoto Encyclopedia of Genes Genomes (KEGG) pathway analysis for genes in each module. The threshold for the analysis was set as count >2 and adjusted *p* < 0.05. Next, we compared the count number of the top 10 significant terms of modules of interest to determine the difference in co-expression patterns between advanced plaque samples and early plaque samples using Wilcoxon’s rank sum test.

### Hub Gene Identification and Crucial Gene Mining

Genes with the highest intramodular connectivity and module membership (MM) >0.8 calculated by the “WGCNA” R package were identified as hub genes for each functional module. Next, the DEG co-expression networks were obtained by mapping DEGs into the whole co-expression network of advanced plaque samples and early plaque samples using Cytoscape v3.7.0. MCODE, a plugin of Cytoscape, was employed to detect the densely connected subnetwork of the DEG co-expression network of advanced plaque samples. The DEG co-expression network of advanced plaque samples and the most significant subnetwork was visualized, and genes with the top 10 degrees in this subnetwork were selected as crucial genes. The MM of the hub genes in the functionally important module was compared between advanced plaque samples and early plaque samples. A DEG co-expression network of the validation set (GSE43292) was also constructed to validate crucial genes. Furthermore, receiver operating characteristic (ROC) analysis was conducted by SPSS 25.0 to demonstrate the potential diagnostic value of these crucial genes to discriminate advanced plaque and early plaque samples.

### LASSO Regression Model and LDA

The lasso algorithm was performed using the R package “glmnet” to prioritize candidate genes in the MCODE subnetwork for building a classifier. The optimal lambda for the coefficient was computed with a minimum value of 10-fold cross-validation in the training set. The dataset was randomly split into training and validation groups at a proportion of 3:1. The LDA model was established and verified using the R package “MASS.”

### Gene Set Enrichment Analysis

GSEA for the 3 crucial genes with the highest degree was conducted using GSEA 4.0.3 software. The c5.bp.v7.0.symbols.gmt and c2.cp.kegg.v7.0.symbols.gmt reference gene sets were downloaded from the official GSEA website^2^. To perform GSEA, the plaque samples were divided into two groups (i.e., samples with high expression levels of crucial genes vs. samples with low expression levels of crucial genes) according to the median value of each gene. Compared with conventional GSEA analysis, the grouping of samples is based on the expression level of each gene in single-gene analysis.

### Sensitivity Analysis of Networks We Constructed

To ensure the robustness of the networks we constructed, we performed a sensitivity analysis. One sample was randomly deleted based on the random number generated by the runif() function in R software. After this sample was deleted, the whole WGCNA pipeline analysis was performed, and crucial genes were identified.

### Statistical Analysis

Data preprocessing, DEG screening, WGCNA and functional and pathway enrichment analysis were performed in R v3.6.2. Crucial genes were mined using MCODE in Cytoscape v3.7.0. GSEA was conducted by GSEA v4.0.3. We described the details of these bioinformatic analyses in the corresponding subsections. The count numbers of the top 10 significant functional terms of modules of interest between advanced plaque and early plaque samples were compared by Wilcoxon’s rank sum test, and the potential diagnostic value of key genes was demonstrated by ROC analysis using IBM SPSS 25.0. The grouping variable of advanced plaque and early plaque (State Variable in SPSS) and the expression value of crucial genes (Test Variable in SPSS) were entered as input, and non-parametric assumptions were chosen as default in ROC analysis. A *p* < 0.05 was considered to be significant.

### Code Availability

The code used in this study (from the GEO datasets to the WGCNA analysis to the crucial gene mining) is available at: https://github.com/cmy2013/classify-early-and-advanced-carotid-plaque.

## Results

### Workflow

**FIGURE 1:**
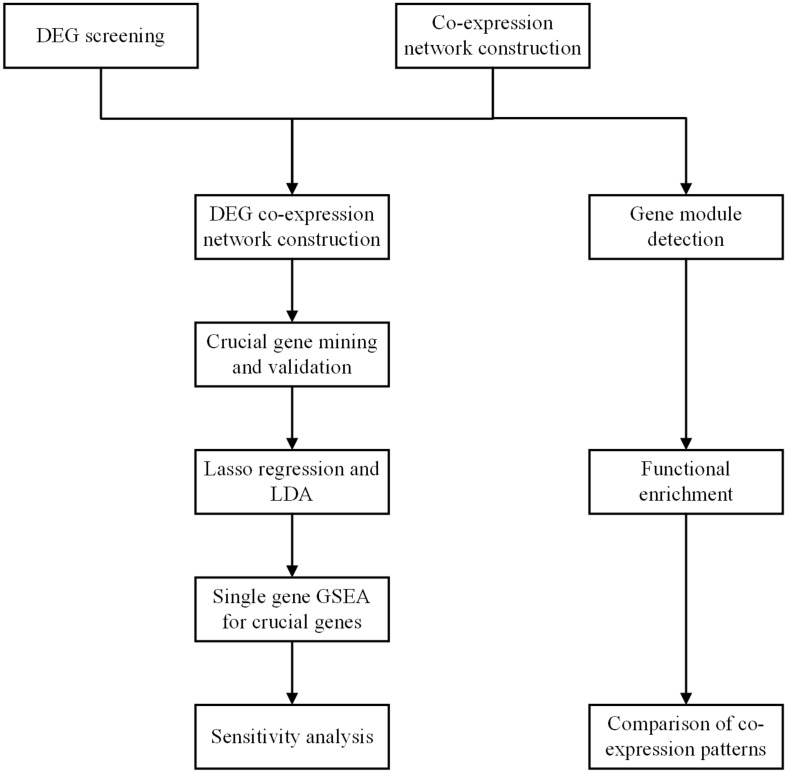
Workflow of the whole study.

The workflow of the present study is shown in Figure 1. DEGs were screened, and the co-expression networks were constructed. A DEG co-expression network was constructed by mapping DEGs into the co-expression network of advanced plaque samples. Crucial genes were obtained based on this DEG co-expression network. These crucial genes were validated based on another independent GEO dataset through the WGCNA pipeline. Next, the genes in the MCODE cluster were used to build a classifier to discriminate advanced plaque and early plaque by lasso regression and LDA analysis. After that step, single-gene GSEA was performed for crucial genes, and their potential clinical significance was determined by ROC analysis. In addition, gene modules were detected for both early plaque and advanced plaque samples. Functional enrichment analysis was conducted using genes in each module. The co-expression patterns were compared between early plaque samples and advanced plaque samples based on the results of functional enrichment analysis. Finally, a sensitivity analysis was performed to evaluate the robustness of the network that we constructed.

### Screening of DEGs

**FIGURE 2:**
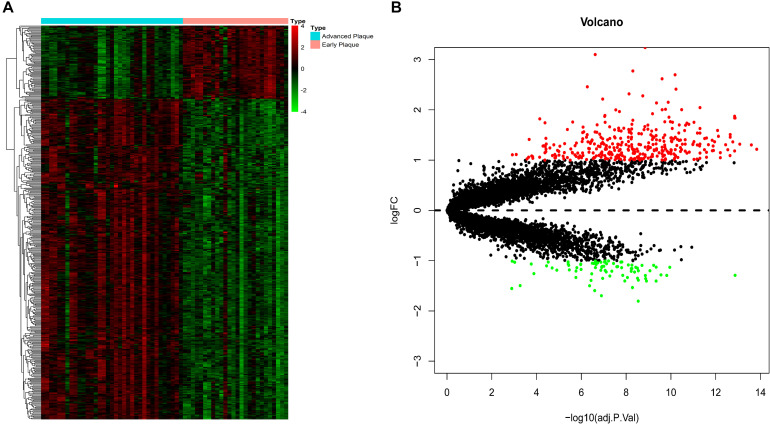
DEG screening. **(A)** Heatmap. The heatmap showed the expression pattern of genes. **(B)** Volcano plot. The x-axis represents the -log_10_(adj.P.Val) while they-axis represents log_2_(fold-change). The red dots represent up-regulated DEGs while the green dots represent the down-regulated DEGs.

The expression patterns of DEGs are shown in Figure 2. With the threshold of adjusted *p* < 0.05 and | log_2_(fold-change)| >1, we screened 436 DEGs, among which 335 were up-regulated and 81 were down-regulated. DEGs with top-10 | log_2_(fold-change)| are shown in Table 1. All DEGs for the training set and validation set are listed in Supplementary Tables S1, S2, respectively. A total of 4,057 overlapping DEGs were identified between the training set and validation set only with the threshold of adjusted *p* < 0.05 (Supplementary Figure S2).

**TABLE 1:** DEGs with top-10 | log_2_(fold-change)| (advance plaque/early plaque).

Gene symbol	Official full name	log_2_(fold-change)	Adjusted *P*-value
**Up-regulated**			
MMP9	Matrix metallopeptidase 9	3.24	1.41E-09
MMP12	Matrix metallopeptidase 12	3.10	2.40E-07
IGJ	Joining chain of multimeric IgA and IgM	2.77	5.01E-09
SPP1	Secreted phosphoprotein 1	2.70	6.52E-11
CHI3L1	Chitinase 3 like 1	2.62	2.48E-10
CCL18	C-C motif chemokine ligand 18	2.46	5.40E-07
APOE	Apolipoprotein E	2.41	5.94E-11
APOC1	Apolipoprotein C1	2.32	7.40E-09
ACP5	Acid phosphatase 5, tartrate resistant	2.28	1.81E-09
MMP7	Matrix metallopeptidase 7	2.21	1.11E-07
**Down-regulated**			
ATP1A2	ATPase Na^+^/K^+^ transporting subunit alpha 2	–1.80	2.87E-09
CNTN1	Contactin 1	–1.70	1.27E-07
MYOCD	Myocardin	–1.59	2.53E-07
ITLN1	Intelectin 1	–1.55	0.001283
CNTN4	Contactin 4	–1.54	5.20E-09
CASQ2	Calsequestrin 2	–1.50	4.28E-07
CARTPT	CART prepropeptide	–1.50	0.00054
BAMBI	BMP and activin membrane bound inhibitor	–1.41	4.57E-10
ACADL	Acyl-CoA dehydrogenase, long chain	–1.41	1.23E-09
PLD5	Phospholipase D family member 5	–1.41	1.15E-08

### Constructed of Weighted Gene Co-expression Network

**FIGURE 3:**
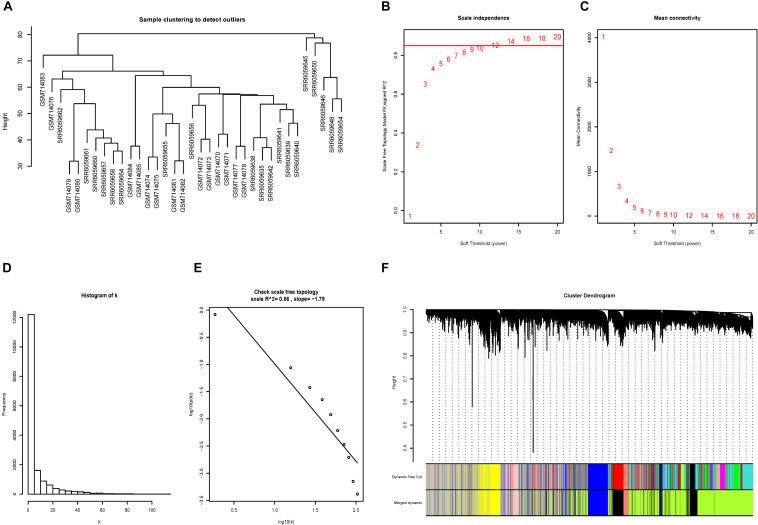
Construction of the co-expression network for advanced plaque. **(A)** No outliers were detected in the sample clustering and all samples were included in further study. **(B,C)** The cut-off for soft-threshold β was set to be 0.85 and β = 14 was selected. **(D,E)** The co-expression network we constructed met the requirements of scale-free topology. **(F)** In advanced plaque sample, 7 gene modules were detected.

No outliers were excluded by sample clustering (Figure 3A and Supplementary Figure S3A). The cut-off of *R*^2^^3^ was set to be 0.85 and soft-threshold power β = 14 and β = 18 were selected for advanced plaque and early plaque samples, respectively (Figures 3B,C and Supplementary Figures S3B,C). The adjacency matrices that store the information of whole co-expression networks were constructed. The histogram and the linear plot showed that both networks that we constructed met the requirements of scale-free topology (Figures 3D,E and Supplementary Figures S3D,E). Next, TOM matrices were created, and gene modules were detected based on TOM matrices. After merging similar modules, seven gene modules were detected for both advanced and early plaque samples (Figure 3F and Supplementary Figure S3F).

### Functional Enrichment of Gene Modules

**FIGURE 4:**
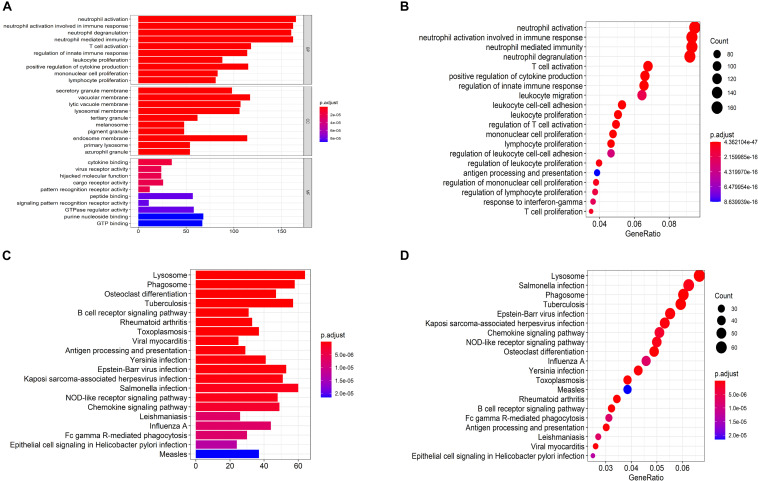
Functional enrichment analysis for GO anal and KEGG pathways for black module of advanced plaque. **(A,B)** Bar plot and dot plot for GO terms of genes in black module. Terms in bar plot and dot plot were ordered by adjusted p value and count number respectively. **(C,D)** Bar plot and dot plot for KEGG pathways of genes in black module. Terms in bar plot and dot plot were ordered by adjusted p value and count number respectively.

The biological functions of the genes in each module were shown by GO-BP and KEGG pathway analysis (Supplementary Tables S3, S4). The co-expression patterns between advanced plaque samples and early plaque samples were compared based on these results. We observed that the black module of advanced plaque samples was associated with inflammation and immune response. GO-BP and KEGG pathways with top-10 significant adjusted p value of this module was shown in Figure 4 and Table 2. The Wilcoxon’s rank sum test (*p* = 0.0003) showed that the count number of black module of advanced plaque samples of these GO-BP and KEGG pathways was significantly larger that of magenta module of early plaque samples, which is the corresponding immune and inflammation associated module in early plaque samples (Supplementary Figure S4). This suggested that genes associated with inflammation and immune response were scattered in the modules of early plaque samples. These results suggested that inflammation and the immune response might play roles in the progression of carotid plaques.

**TABLE 2:** GO-BP and KEGG pathway terms with top-10 significant adjusted *P*-value for black module of advanced plaque samples.

Terms	Count	Adjusted *p*-value
**GO-BP**		
Neutrophil activation	165	4.36E-47
Neutrophil activation involved in immune response	162	1.38E-46
Neutrophil degranulation	160	9.85E-46
Neutrophil mediated immunity	162	2.03E-45
T cell activation	118	1.10E-21
Regulation of innate immune response	114	1.24E-20
Leukocyte proliferation	88	1.55E-20
Positive regulation of cytokine production	115	2.82E-20
Mononuclear cell proliferation	83	3.50E-20
Lymphocyte proliferation	81	3.55E-19
**KEGG pathways**		
Lysosome	64	4.38E-24
Phagosome	58	6.53E-15
Osteoclast differentiation	47	2.43E-11
Tuberculosis	57	7.38E-11
B cell receptor signaling pathway	31	8.04E-08
Rheumatoid arthritis	33	1.21E-07
Toxoplasmosis	37	1.21E-07
Viral myocarditis	25	1.94E-07
Antigen processing and presentation	29	2.40E-07
Yersinia infection	41	3.18E-07

### Identification of Hub Genes and Mining of Crucial Genes

The intramodular connectivity for genes in each module was calculated by the WGCNA algorithm. Genes with the highest intramodular connectivity and MM >0.8 were selected as the hub genes of each module (Supplementary Table S5). *ARHGAP18* (Rho GTPase activating protein 18) was the hub gene of the black module in advanced plaque samples. The MM (0.92) of *ARHGAP18* in black module of advanced plaque was larger than MM (0.79) of *ARHGAP18* in corresponding module (purple) of early plaque.

**FIGURE 5:**
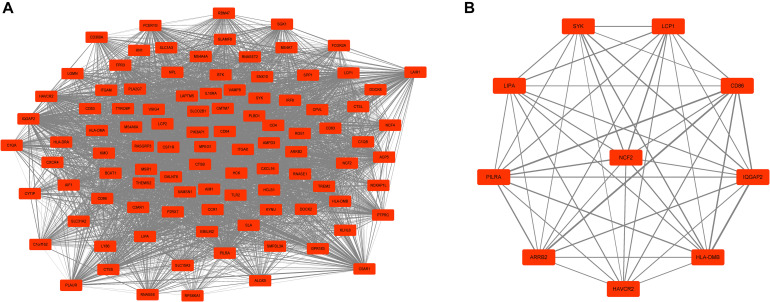
MCODE cluster and crucial genes. **(A)** Subnetwork of the most significant MCODE cluster. The red boxes represent the up-regulated genes while the green boxes represent down-regulated genes. **(B)** Genes with top-10 degree were considered as crucial genes. *NCF2*, *IQGAP2*, and *CD86* were the genes with the highest degree among these crucial genes.

**FIGURE 6:**
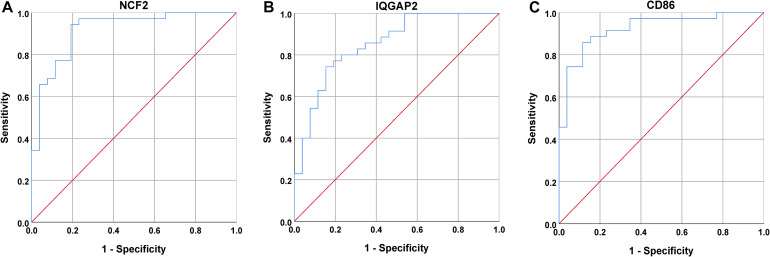
ROC curves for *NCF2, IQGAP2*, and *CD86*. **(A)** The AUC for NCF2 was 0.918. **(B)** The AUC for IQGAP2 was 0.853. **(C)** The AUC for CD86 was 0.903.

**FIGURE 7:**
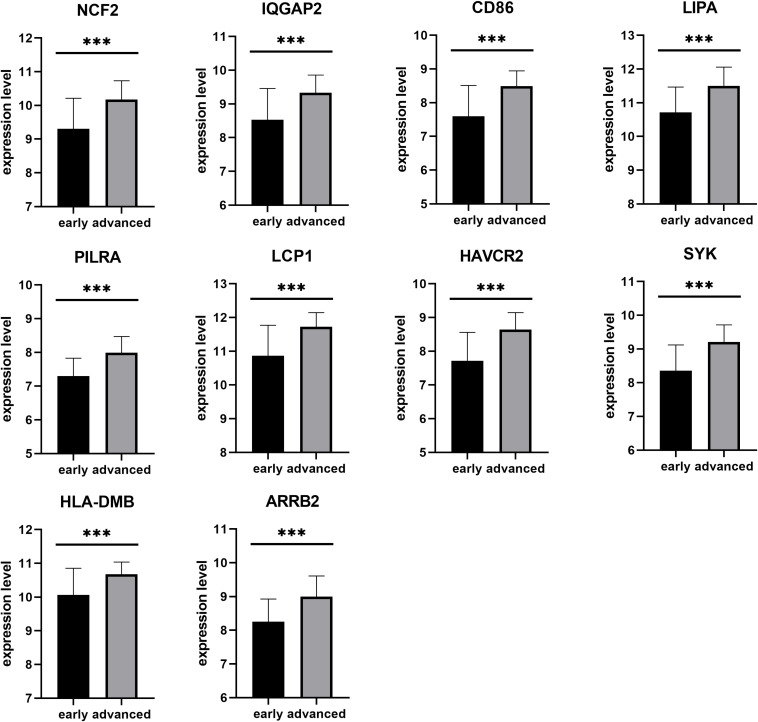
Validation of crucial genes by GSE43292. All crucial genes were found in the DEG co-expression network of the validation set (***p < 0.001).

Next, a DEG co-expression network was generated by mapping DEGs into the whole co-expression network of advanced plaques. After removing isolated nodes and node pairs, the network had 393 edges and 344 nodes (Supplementary Figure S5). The MCODE subnetwork and 10 crucial genes were visualized (Figure 5). *NCF2* (neutrophil cytosolic factor 2), *IQGAP2* (IQ motif containing GTPase activating protein 2) and *CD86* (CD86 molecule) were the genes with the highest degree in the crucial gene cluster. ROC analysis was performed, and all these crucial genes showed potential diagnostic significance. The areas under the curve (AUCs) of most crucial genes were above 0.90 (Table 3). The ROC curves of *NCF2, IQGAP2*, and *CD86* are visualized in Figure 6. All these crucial genes were found in the DEG co-expression network of GSE43292 and were successfully validated (Figure 7 and Supplementary Table S6).

**TABLE 3:** AUC for crucial genes.

Gene symbol	Official full name	AUC	*p*-value
NCF2	Neutrophil cytosolic factor 2	0.918	<0.001
IQGAP2	IQ motif containing GTPase activating protein 2	0.853	<0.001
CD86	CD86 molecule	0.903	<0.001
LIPA	Lipase A, lysosomal acid type	0.923	<0.001
PILRA	Paired immunoglobin like type 2 receptor alpha	0.948	<0.001
LCP1	Lymphocyte cytosolic protein 1	0.948	<0.001
HAVCR2	Hepatitis A virus cellular receptor 2	0.937	<0.001
SYK	Spleen associated tyrosine kinase	0.957	<0.001
HLA-DMB	Major histocompatibility complex, class II, DM beta	0.927	<0.001
ARRB2	Arrestin beta 2	0.91	<0.001

### LASSO Regression Analysis and LDA

**FIGURE 8:**
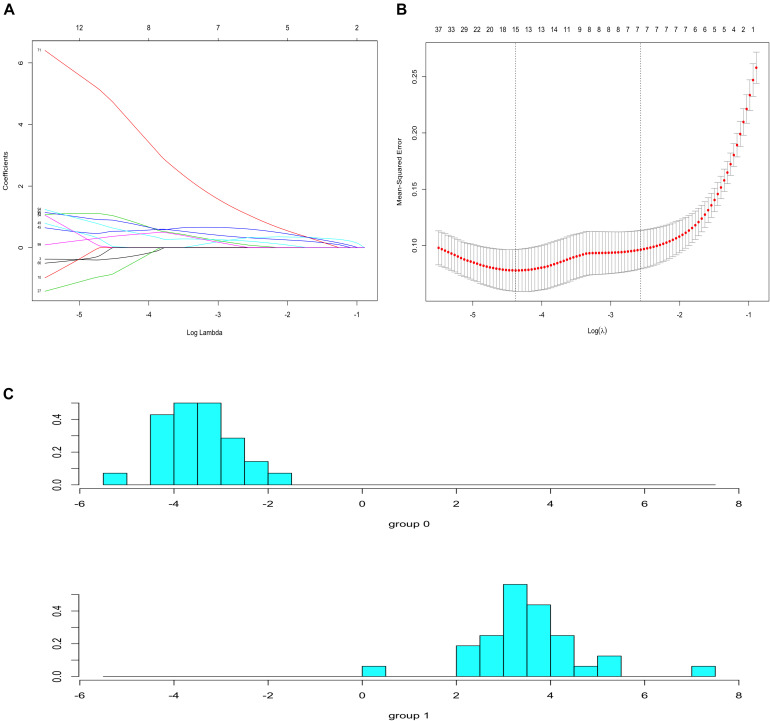
Lasso regression and LDA analysis. **(A)** Lasso coefficient profiles of 100 genes. **(B)** 10-fold cross-validation for selecting minimal λ based on 1-SE criteria for recurrence. A total of 15 genes were selected. **(C)** There was a clear shift in LDA function, with a left shift being observed for early plaques and a right shift for advanced plaques.

To identify prognostic markers in the progression of atherosclerosis, the top 100 genes in the MCODE subnetwork were input into the LASSO regression model. The lambda value was 0.0125, and 15 genes were selected to calculate the risk score (Figures 8A,B and Supplementary Table S7. The risk model was constructed with the coefficients of 15 genes: *IQGAP2*, *FPR3*, *FCER1G*, *SLC1A3*, *C5AR1*, *PLA2G7*, *ALOX5*, *CCR1*, *RASGRP3*, *SLAMF8*, *C3AR1*, *AIF1*, *AMPD3*, *BTK*, and *CTSB*. There was a clear shift in LDA function, with a left shift being observed for early plaques and a right shift for advanced plaques. The accuracy of the LDA model using the confusion matrix algorithm was 100% (Figure 8C). These results indicated that the classifier can clearly discriminate advanced plaque and early plaque.

### Gene Set Enrichment Analysis

**FIGURE 9:**
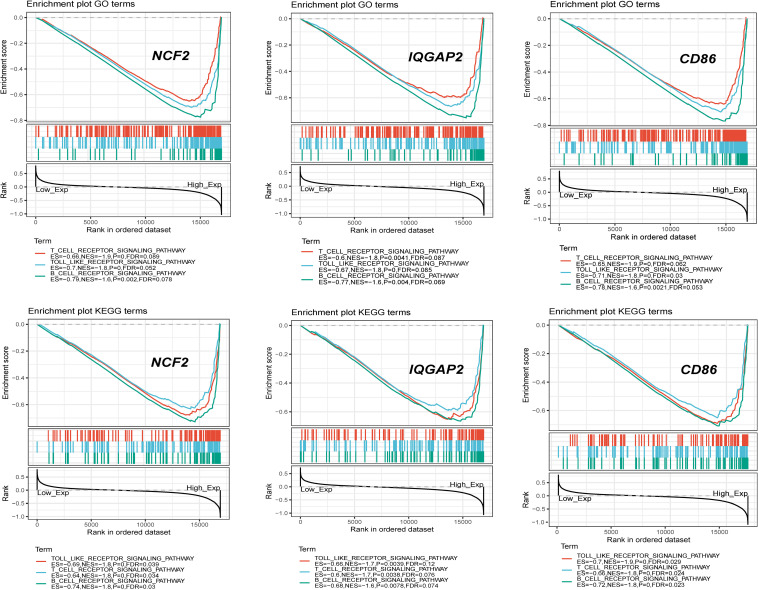
Single gene GSEA for 3 crucial genes with highest degree.

Single-gene GSEA for each crucial gene was performed. Gene sets associated with the immune response and inflammation were highly up-regulated in the group with high expression levels of crucial genes (e.g., the *NCF2*-high group), which also suggested that the immune response and inflammation play important roles in the progression of carotid atherosclerosis (Figure 9).

### Sensitivity Analysis of Constructed Network

We ranked the sample ID in ascending order. Next, the runif() function generated 18 and 13 as random numbers for advanced plaque samples and early plaque samples. The corresponding samples (SRR6059638 for advanced plaque and GSM714098 for early plaque samples) were deleted. The results of sample clustering (Supplementary Figures S6A, S7A) were nearly the same as those of the original network construction (Figure 3A and Supplementary Figure S3A), except the deleted samples were not in the results. Then, with the same cutoff, the same soft-threshold βs (β = 14 for advanced plaque samples and β = 18 for early plaque samples) were selected to construct co-expression network (Supplementary Figures S3B–E, S6B–E). In the module detection, the same numbers of modules (7 gene modules) were detected for both groups (Supplementary Figures S6F, S7F). Finally, most of the crucial genes overlapped with the original analysis, except that *ARRB2* was replaced by *ACP5* (Supplementary Figure S8), which was also in the MCODE cluster. The results of the sensitivity analysis showed that our results were reliable.

## Discussion

In the present study, 436 DEGs were identified in plaque samples. Based on weighted gene co-expression networks and modules were constructed using the WGCNA algorithm. Functional enrichment analyses of GO and KEGG were performed for each module. In the GO-BP and KEGG pathway analyses, pathways associated with inflammation and immune response were clustered in the black module of advanced plaque samples. These pathways were dispersed in early plaque samples. *NCF2, IQPAG2*, and *CD86* had the highest connectivity among the crucial genes identified by MCODE. Based on single gene GSEA, gene sets correlated with inflammation and immune response were highly up-regulated in the group of high expression level of crucial genes. Finally, the sensitivity analysis showed that our results were reliable.

Several previous studies have used the microarray dataset GSE28829 to screen DEGs (Lin et al., 2014; Wang et al., 2014; Tan et al., 2017; Liu et al., 2018). Different methods and different criteria lead to differences in DEG results. Wang et al. screened 267 up-regulated genes and 52 down-regulated genes. The hub gene was TYRO protein tyrosine kinase binding protein (*TYROBP*). CD14, which is one of the crucial genes in the present study, was one of up-regulated the up-regulated genes revealed by COXPRESdb (Wang et al., 2014). Liu et al. obtained 758 differentially expressed genes with thresholds of FDR <0.05 and | log_2_FC| >0.58. *ITGAM* and *ACTN2* have the highest degree in the PPI network (Liu et al., 2018). Lin et al. detected DEGs by robust multiarray average (RMA). In the study by these researchers, 322 up-regulated genes and 385 down-regulated genes were identified in advanced plaque samples. *CFL2* and *MMP9* were identified as important genes in the transcriptional regulatory network (Lin et al., 2014). Tan et al. used the signal-to-noise method and listed the top 100 up-regulated genes and top 100 down-regulated genes. *IGK, MMP9*, and *IGLC2* are hub genes for up-regulated genes. *AW451999, CFL2*, and *PDZRN3* are hub genes for down-regulated genes (Tan et al., 2017). Other datasets were also employed to identify the DEGs. Liu et al. (2020) analyzed GSE41571, GSE118481, and E-MTAB-2055 and discovered that *JCHAIN, CXCL10*, and *HMOX1* were the crucial genes for up-regulated genes, and the most down-regulated genes were *COL21A1, DACT3*, and *ACTC1* in unstable plaques. However, these studies did not use a weighted and scale-free network approach, which identifies genes with critical positions in the whole network, to elucidate the molecular mechanism governing plaque progression.

Some studies have identified key genes in atherosclerosis progression using WGCNA. By applying lncRNA classification to the GSE28829 dataset and confirming correlated genes by conducting WGCNA to analyze GSE40231, Wang et al. identified 6 lncRNAs, including *ZFAS1* and *LOC100506730*, that play crucial roles in atherosclerosis. These lncRNAs are involved in the interferon-gamma-mediated signaling pathway and leukotriene biosynthetic process (Wang C.H. et al., 2019). Albright et al. (2014) discovered that pathways that regulate inflammatory and macrophage gene function were correlated with atherosclerosis, and *CD44* was a critical gene.

The present study compared the co-expression patterns between early and advanced plaques using the WGCNA method and generated a DEG co-expression network by mapping DEGs into the whole co-expression network of advanced plaque samples for the first time.

The functional enrichment of WGCNA modules indicated that inflammation and immune response are related to the progression of atherosclerosis. Atherosclerosis is overwhelmingly proven to be a chronic inflammatory disease (Hansson et al., 2006; Wolf and Ley, 2019). Bioinformatic, experimental, clinical and epidemiological studies demonstrated a relationship between inflammation and lipid metabolism. After low-density lipoproteins deposit in the intima, monocytes are recruited by leukocyte adhesion molecules and differentiate into macrophages. By upregulating scavenger receptors and Toll-like receptors, macrophages mediate lipoprotein internalization and transmit activating signals. Severe inflammation may lead to plaque rupture and thrombosis. Inflammatory markers, including CRP, IL-6, and Pentraxin-3, are used to monitor the atherosclerosis process in coronary artery disease (Hansson et al., 2006). The immune response is initiated by specific antigens, including the ApoB100 protein of LDL. Regulatory T cells secrete atheroprotective IL-10 and TGF-β to attenuate atherosclerosis, while Th1 cells promote atherosclerosis. As atherosclerosis progresses, T cells express proatherogenic Treg transcription factors, including RORγ-T (Th17), Bcl-6 (TFH), or T-bet (Th1), instead of the atheroprotective Th transcription factor FoxP3 (Gistera and Hansson, 2017). This finding is consistent with the fraction of regulatory T cells in plaque samples that we calculated by the CIBERSORT algorithm.

The hub gene of the black module was *ARHGAP18*. *ARHGAP18* (SENEX) is a negative regulator of YAP and RhoC (Coleman et al., 2020). Overexpression of RhoC destroys the actin cytoskeleton and cell junctions. *ARHGAP18* regulates the alignment of endothelial cells and transmigration of neutrophils by stabilizing microtubes (Lovelace et al., 2017). Lay et al. (2019) reported that the expression of *ARHGAP18* increased in areas where plaques formed and progressed. This finding is in keeping with our conclusion. Areas with turbulence and low shear force, such as carotid artery bifurcation, are high-risk areas for arterial plaque formation. In *in vitro* experiments, the expression of *ARHGAP18* increased in areas with low wall shear stress and turbulence, which are risk factors for atherosclerosis.

Among these crucial genes detected by MCODE based on the DEG co-expression network, *NCF2, IQGAP2*, and *CD86* had the highest connectivity. ROC curve analysis of these three genes was performed. The AUC was 0.918 for *NCF2*, 0.853 for *IQGAP2*, and 0.923 for *CD86.*

*NCF2* encoded a 526-amino acid protein P67^phox^ (de Albuquerque et al., 2019). Recent reports demonstrated that *NCF2* was significantly associated with hypertension by increasing the expression and activity of NOXs and reactive oxygen species (ROS) generation (Risley et al., 2003; Li et al., 2018). Bioinformatic analyses indicated that up-regulated expression of *NCF2* increased susceptibility to unstable atherosclerotic plaque-related stroke (Zhou et al., 2020). Few studies, however, have evaluated the potential mechanism of the correlation between the *NCF2* variant and the progression of atherosclerosis. P67^phox^ is a subunit of NADPH oxidase 2 (NOX2). NOX2 is up-regulated in unstable atherosclerotic plaques (Singel and Segal, 2016). NOX2 activity contributes to the formation and accumulation of oxidized low-density lipoproteins (ox-LDL) via ROS products (Violi et al., 2017). The association between NOX2 and atherosclerosis was substantiated by mouse knockouts lacking subunits of Nox2. Inhibition of NOX2 by pharmacological intervention also delayed the atherosclerotic process. These results indicated that *NCF2* might mediate the progression of atherosclerosis through NOX2.

*IQGAP2* expresses IQ motif-containing GTPase-activating protein 2, which is a 180-kDa multidomain scaffolding protein (Ghaleb et al., 2015). *IQGAP2* was first classified as a tumor suppressor expressed in the liver and was reported to regulate the PI3K/Akt and Wnt/β-catenin signaling pathways (Gnatenko et al., 2013). The PI3K/Akt pathway was demonstrated to be associated with polarization and apoptosis of macrophages by controlling mTOR assembly (Linton et al., 2016). Seimon and Tabas (2009) reported that stimulating macrophage apoptosis decreased atherosclerotic lesions in early plaques and that suppressing apoptosis increased atherosclerosis. In addition, the role of *IQGAP2* in diminishing the production and recruitment of macrophages was evidenced in animal experiments. *IQGAP2* has been shown to bind Rho GTPase rac1/cdc42 by the GTPase-binding domain in platelets and thus regulate NF-κB signaling (Schmidt et al., 2003). The signaling of Toll-like receptor 4 (TLR4)/NF-κB was impaired in Iqgap2^–/–^ mice. Interleukin-6 (IL-6), a pro-inflammatory cytokine driven by NF-κB, was subsequently suppressed. Various studies have demonstrated that IL-6 is essential in the downstream inflammatory response that mediates the initiation and progression of atherosclerosis (Wang et al., 2017).

*CD86* expression and the M1/M2 marker ratio (CD86/CD163) were significantly higher in vulnerable plaques than in stable plaques (Williams et al., 2017). Deposited oxidized lipoproteins activate scavenger receptors, such as *CD36* and *TLR4*, to polarize macrophages to the M1 subtype and eventually form foam cells (Momtazi-Borojeni et al., 2019). M1 phenotype macrophages release ROS and proinflammatory cytokines, such as TNF-α, IL-1β, IL-6, and IL-12, that damage endothelial cells and vessels (Shapouri-Moghaddam et al., 2018). In addition to M1 macrophages, *CD86* was also found to be expressed on mature dendritic cells and partial T cells (Ewing et al., 2013). The expression of *CD86* on dendritic cells was shown to be up-regulated in patients with coronary artery disease (Dopheide et al., 2007). In *CD80*^–/–^*CD86*^–/–^mice, the progression of atherosclerosis was delayed. Vignali et al. (2008) reported that *CD80/86* dendritic cells play roles in the co-stimulation of Th1 cells. The proatherogenic role of Th1 cells has been clearly evidenced in a large body of animal experimental studies (Saigusa et al., 2020). T-cell and *CD28*-*CD80/86* co-stimulation plays a vital role in both plaque formation and atherosclerosis development (Zirlik and Lutgens, 2015).

In addition to the top 3 crucial genes, other genes, such as the *LIPA* gene, also play important roles in atherosclerosis by encoding lysosomal acid lipase (LAL). Depletion of *LIPA* causes lipid metabolism disorders in mice (Li and Zhang, 2019). Bioinformatic studies identified that *LIPA* is correlated with coronary heart disease (Mehta, 2011; Nelson et al., 2017). Lysosomal acid lipase increased the release and degradation of triglycerides and cholesteryl esters in macrophages and hepatocytes (Du and Grabowski, 2004). The fatty acid liberated by hydrolysis elevated the expression of peroxisome proliferator-activated receptor-γ, which up-regulated the production of *CD36* and consequently enhanced the uptake of oxLDL (Chistiakov et al., 2016). Further studies are necessary to elucidate how lysosomal acid lipase contributes to atherosclerosis.

Single-gene GSEA indicated that crucial genes were significantly associated with immune and inflammatory pathways. The most frequent pathways related to crucial genes were B cell receptor signaling, the cell cycle, T cell receptor signaling and the Toll-like receptor signaling pathway. *NCF2* plays key roles in the TLR4/NOX2 signaling pathway by expressing the subunit of NOX2 (Wang B. et al., 2019). *IQGAP2* regulates TLR4/NF-κB signaling by binding Rho GTPase rac1/cdc42. The activation of the Toll-like receptor upregulates the expression of *CD86* in macrophages and T cells. The results of this study confirmed the biological significance of this method for screening crucial genes.

In this study, we constructed a DEG co-expression network between early carotid plaques and advanced plaques for the first time. The hub genes and crucial genes were identified. *NCF2, IQGAP2*, and *CD86* might play crucial roles in the process of carotid atherosclerosis. However, our study has several limitations. These genes have potential value for the diagnosis and treatment of carotid plaques. The GSE28829 and GSE104140 datasets lack clinical information. We cannot correlate clinical traits with gene modules. The results were based on data downloaded from GEO datasets, and further studies are needed to explore the detailed molecular mechanism of carotid atherosclerosis progression *in vitro* and *in vivo*.

## Data Availability Statement

The datasets presented in this study can be found in online repositories. The names of the repository/repositories and accession number(s) can be found below: https://www.ncbi.nlm.nih.gov/, GSE28829; https://www.ncbi.nlm.nih.gov/, GSE43292; https://www.ncbi.nlm.nih.gov/, GSE104140.

## Author Contributions

MC designed experiments, analyzed data, and wrote the manuscript. SC designed experiments, prepared figures and tables, and wrote the manuscript. DY devised the concept, analyzed the data, and revised the manuscript. JZ, BL, YC, and WY designed experiments and edited the manuscript. HZ and LJ analyzed data and wrote the manuscript. YZ devised the concept, designed the research, supervised the study, and edited the manuscript. All authors contributed to the article and approved the submitted version.

## Conflict of Interest

The authors declare that the research was conducted in the absence of any commercial or financial relationships that could be construed as a potential conflict of interest.

## Footnotes

1 https://www.ncbi.nlm.nih.gov/geo/

2 https://www.gsea-msigdb.org/gsea/downloads.jsp

3 https://www.ncbi.nlm.nih.gov/sra/

## Supplementary Material

Supplementary Figure 1 Batch normalization between GSE28829 and GSE104140.

Supplementary Figure 2 Overlapping of all DEGs between training set and validation. The only threshold was set as adjusted *p* < 0.05.

Supplementary Figure 3 Construction of the co-expression network for early plaque. **(A)** No outliers were detected in the sample clustering and all samples were included in further study. **(B,C)** The cut-off for soft-threshold β was set to be 0.85 and β = 18 was selected. **(D,E)** The co-expression network we constructed met the requirements of scale-free topology. **(F)** In early plaque sample, 7 gene modules were detected.

Supplementary Figure 4 Functional enrichment analysis for GO anal and KEGG pathways for magenta module of early plaque. **(A,B)** Bar plot and dot plot for GO terms of genes in magenta module. Terms in bar plot and dot plot were ordered by adjusted *p* value and count number respectively. **(C,D)** Bar plot and dot plot for KEGG pathways of genes in magenta module. Terms in bar plot and dot plot were ordered by adjusted *p* value and count number, respectively.

Supplementary Figure 5 DEG co-expressionnetwork. The red boxes represent the up-regulated genes while the green boxes represent down-regulated genes.

Supplementary Figure 6 Construction of the co-expression network for advanced plaque in sensitivity analysis. SRR6059638 was delete based on generated random number. **(A)** No outliers were detected in the sample clustering and all samples were included in further study. **(B,C)** The cut-off for soft-threshold β was set to be 0.85 and β = 14 was selected. **(D,E)** The co-expression network we constructed met the requirements of scale-free topology. **(F)** In this sensitivity analysis of advanced plaque sample, 7 gene modules were detected, which is the same as that of the original network construction of advanced plaque.

Supplementary Figure 7 Construction of the co-expression network for early plaque in sensitivity analysis. GSM714098 was delete based on generated random number. **(A)** No outliers were detected in the sample clustering and all samples were included in further study. **(B,C)** The cut-off for soft-threshold β was set to be 0.85 and β = 18 was selected. **(D,E)** The co-expression network we constructed met the requirements of scale-free topology. **(F)** In this sensitivity analysis of early plaque sample, 7 gene modules were detected, which is the same as that of the original network construction of early plaque.

Supplementary Figure 8 Crucial genes identified in sensitivity analysis. Most of the crucial genes overlapped with the original analysis, except that ARRB2 was replaced by ACP5

Supplementary Table 1 DEGs of training set under the threshold of adjusted *p* < 0.05.

Supplementary Table 2 DEGs of validation set under the treshold of adjusted *p* < 0.05.

Supplementary Table 3 GO terms of gene modules for advanced plaque and early plaque.

Supplementary Table 4 KEGG pathways of gene modules for advanced plaque and early plaque.

Supplementary Table 5 Hub genes for each module of early plaque and advanced plaque.

Supplementary Table 6 Genes in DEG co-expression network of validation set.

Supplementary Table 7 Coefficients of lasso regression.

## References

[B1] Albright J. Quizon P. M. Lusis A. J. Bennett B. J. (2014). Genetic network identifies novel pathways contributing to atherosclerosis susceptibility in the innominate artery. *BMC Med. Genomics* 7:51. 10.1186/1755-8794-7-51 25115202 PMC4142055

[B2] Alloza I. Goikuria H. Idro J. L. Trivino J. C. Fernandez Velasco J. M. Elizagaray E. (2017). RNAseq based transcriptomics study of SMCs from carotid atherosclerotic plaque: BMP2 and IDs proteins are crucial regulators of plaque stability. *Sci. Rep.* 7:3470. 10.1038/s41598-017-03687-9 28615715 PMC5471186

[B3] Chen S. Yang D. Lei C. Li Y. Sun X. Chen M. (2019). Identification of crucial genes in abdominal aortic aneurysm by WGCNA. *PeerJ* 7:e7873. 10.7717/peerj.7873 31608184 PMC6788446

[B4] Chistiakov D. A. Bobryshev Y. V. Orekhov A. N. (2016). Macrophage-mediated cholesterol handling in atherosclerosis. *J. Cell Mol. Med.* 20 17–28. 10.1111/jcmm.12689 26493158 PMC4717859

[B5] Coleman P. R. Lay A. J. Ting K. K. Zhao Y. Li J. Jarrah S. (2020). YAP and the RhoC regulator ARHGAP18, are required to mediate flow-dependent endothelial cell alignment. *Cell Commun. Signal.* 18:18 10.1186/s12964-020-0511-7 PMC6998144 32013974

[B6] de Albuquerque J. A. T. Lima A. M. de Oliveira Junior E. B. Ishizuka E. K. Aragão-Filho W. C. Bengala Zurro N. (2019). A novel mutation in the NCF2 gene in a CGD patient with chronic recurrent pneumopathy. *Front. Pediatr.* 7:391. 10.3389/fped.2019.00391 31612120 PMC6776604

[B7] Dobin A. Davis C. A. Schlesinger F. Drenkow J. Zaleski C. Jha S. (2013). STAR: ultrafast universal RNA-seq aligner. *Bioinformatics* 29 15–21. 10.1093/bioinformatics/bts635 23104886 PMC3530905

[B8] Dopheide J. F. Sester U. Schlitt A. Horstick G. Rupprecht H. J. Münzel T. (2007). Monocyte-derived dendritic cells of patients with coronary artery disease show an increased expression of costimulatory molecules CD40, CD80 and CD86 in vitro. *Coron. Artery Dis.* 18 523–531. 10.1097/MCA.0b013e3282eff1ad 17925605

[B9] Du H. Grabowski G. A. (2004). Lysosomal acid lipase and atherosclerosis. *Curr. Opin. Lipidol.* 15 539–544. 10.1097/00041433-200410000-00007 15361789

[B10] Ewing M. M. Karper J. C. Abdul S. de Jong R. C. Peters H. A. de Vries M. R. (2013). T-cell co-stimulation by CD28-CD80/86 and its negative regulator CTLA-4 strongly influence accelerated atherosclerosis development. *Int. J. Cardiol.* 168 1965–1974. 10.1016/j.ijcard.2012.12.085 23351788

[B11] Franceschini N. Giambartolomei C. de Vries P. S. Finan C. Bis J. C. Huntley R. P. (2018). GWAS and colocalization analyses implicate carotid intima-media thickness and carotid plaque loci in cardiovascular outcomes. *Nat. Commun.* 9:5141. 10.1038/s41467-018-07340-5 30510157 PMC6277418

[B12] Ghaleb A. M. Bialkowska A. B. Snider A. J. Gnatenko D. V. Hannun Y. A. Yang V. W. (2015). IQ Motif-containing GTPase-activating protein 2 (IQGAP2) is a novel regulator of colonic inflammation in mice. *PLoS One* 10:e0129314. 10.1371/journal.pone.0129314 26047140 PMC4457730

[B13] Gistera A. Hansson G. K. (2017). The immunology of atherosclerosis. *Nat. Rev. Nephrol.* 13 368–380. 10.1038/nrneph.2017.51 28392564

[B14] Gnatenko D. V. Xu X. Zhu W. Schmidt V. A. (2013). Transcript profiling identifies iqgap2(-/-) mouse as a model for advanced human hepatocellular carcinoma. *PLoS One* 8:e71826. 10.1371/journal.pone.0071826 23951254 PMC3741273

[B15] Golledge J. Greenhalgh R. M. Davies A. H. (2000). The symptomatic carotid plaque. *Stroke* 31 774–781. 10.1161/01.str.31.3.774 10700518

[B16] Hansson G. K. Robertson A. K. Soderberg-Naucler C. (2006). Inflammation and atherosclerosis. *Annu. Rev. Pathol.* 1 297–329. 10.1146/annurev.pathol.1.110304.100100 18039117

[B17] Lay A. J. Coleman P. R. Formaz-Preston A. Ting K. K. Roediger B. Weninger W. (2019). ARHGAP18: a flow-responsive gene that regulates endothelial cell alignment and protects against atherosclerosis. *J. Am. Heart Assoc.* 8:e010057. 10.1161/jaha.118.010057 30630384 PMC6497359

[B18] Li F. Zhang H. (2019). Lysosomal acid lipase in lipid metabolism and beyond. *Arterioscler. Thromb. Vasc. Biol.* 39 850–856. 10.1161/atvbaha.119.312136 30866656 PMC6482091

[B19] Li H. Han X. Hu Z. Huang J. Chen J. Hixson J. E. (2018). Associations of NADPH oxidase-related genes with blood pressure changes and incident hypertension: the gensalt study. *J. Hum. Hypertens* 32 287–293. 10.1038/s41371-018-0041-6 29463833 PMC5889722

[B20] Libby P. Ridker P. M. Hansson G. K. (2011). Progress and challenges in translating the biology of atherosclerosis. *Nature* 473 317–325. 10.1038/nature10146 21593864

[B21] Lin M. Zhao L. Zhao W. Weng J. (2014). Dissecting the mechanism of carotid atherosclerosis from the perspective of regulation. *Int. J. Mol. Med.* 34 1458–1466. 10.3892/ijmm.2014.1960 25318463 PMC4214333

[B22] Linton M. F. Babaev V. R. Huang J. Linton E. F. Tao H. Yancey P. G. (2016). Macrophage apoptosis and efferocytosis in the pathogenesis of atherosclerosis. *Circ. J.* 80 2259–2268. 10.1253/circj.CJ-16-0924 27725526 PMC5459487

[B23] Liu J. Jing L. Tu X. (2016). Weighted gene co-expression network analysis identifies specific modules and hub genes related to coronary artery disease. *BMC Cardiovasc. Disord.* 16:54. 10.1186/s12872-016-0217-3 26944061 PMC4779223

[B24] Liu W. Zhao Y. Wu J. (2018). Gene expression profile analysis of the progression of carotid atherosclerotic plaques. *Mol. Med. Rep.* 17 5789–5795. 10.3892/mmr.2018.8575 29436628 PMC5866022

[B25] Liu Y. Huan W. Wu J. Zou S. Qu L. (2020). IGFBP6 is downregulated in unstable carotid atherosclerotic plaques according to an integrated bioinformatics analysis and experimental verification. *J. Atheroscler. Thromb.* 27 1068–1085. 10.5551/jat.52993 32037372 PMC7585910

[B26] Lovelace M. D. Powter E. E. Coleman P. R. Zhao Y. Parker A. Chang G. H. (2017). The RhoGAP protein ARHGAP18/SENEX localizes to microtubules and regulates their stability in endothelial cells. *Mol. Biol. Cell* 28 1066–1078. 10.1091/mbc.E16-05-0285 28251925 PMC5391183

[B27] Mehta N. N. (2011). A genome-wide association study in europeans and South asians identifies 5 new Loci for coronary artery disease. *Circ. Cardiovasc. Genet.* 4 465–466. 10.1161/circgenetics.111.960989 21846871 PMC3190399

[B28] Mo X. G. Liu W. Yang Y. Imani S. Lu S. Dan G. (2019). NCF2, MYO1F, S1PR4, and FCN1 as potential noninvasive diagnostic biomarkers in patients with obstructive coronary artery: a weighted gene co-expression network analysis. *J. Cell Biochem.* 120 18219–18235. 10.1002/jcb.29128 31245869 PMC6771964

[B29] Momtazi-Borojeni A. A. Abdollahi E. Nikfar B. Chaichian S. Ekhlasi-Hundrieser M. (2019). Curcumin as a potential modulator of M1 and M2 macrophages: new insights in atherosclerosis therapy. *Heart Fail. Rev.* 24 399–409. 10.1007/s10741-018-09764-z 30673930

[B30] Nelson C. P. Goel A. Butterworth A. S. Kanoni S. Webb T. R. Marouli E. (2017). Association analyses based on false discovery rate implicate new loci for coronary artery disease. *Nat. Genet.* 49 1385–1391. 10.1038/ng.3913 28714975

[B31] Newman A. M. Liu C. L. Green M. R. Gentles A. J. Feng W. Xu Y. (2015). Robust enumeration of cell subsets from tissue expression profiles. *Nat. Methods* 12 453–457. 10.1038/nmeth.3337 25822800 PMC4739640

[B32] Perisic L. Aldi S. Sun Y. Folkersen L. Razuvaev A. Roy J. (2016). Gene expression signatures, pathways and networks in carotid atherosclerosis. *J. Intern. Med.* 279 293–308. 10.1111/joim.12448 26620734

[B33] Risley P. Jerrard-Dunne P. Sitzer M. Buehler A. von Kegler S. Markus H. S. (2003). Promoter polymorphism in the endotoxin receptor (CD14) is associated with increased carotid atherosclerosis only in smokers: the carotid atherosclerosis progression study (CAPS). *Stroke* 34 600–604. 10.1161/01.Str.0000055941.61801.5a 12624278

[B34] Saigusa R. Winkels H. Ley K. (2020). T cell subsets and functions in atherosclerosis. *Nat. Rev. Cardiol.* 17 387–401. 10.1038/s41569-020-0352-5 32203286 PMC7872210

[B35] Schmidt V. A. Scudder L. Devoe C. E. Bernards A. Cupit L. D. Bahou W. F. (2003). IQGAP2 functions as a GTP-dependent effector protein in thrombin-induced platelet cytoskeletal reorganization. *Blood* 101 3021–3028. 10.1182/blood-2002-09-2807 12515716

[B36] Seimon T. Tabas I. (2009). Mechanisms and consequences of macrophage apoptosis in atherosclerosis. *J. Lipid. Res.* 50(Suppl.), S382–S387. 10.1194/jlr.R800032-JLR200 18953058 PMC2674693

[B37] Shapouri-Moghaddam A. Mohammadian S. Vazini H. Taghadosi M. Esmaeili S. A. Mardani F. (2018). Macrophage plasticity, polarization, and function in health and disease. *J. Cell Physiol.* 233 6425–6440. 10.1002/jcp.26429 29319160 PMC13482560

[B38] Singel K. L. Segal B. H. (2016). NOX2-dependent regulation of inflammation. *Clin. Sci.* 130 479–490. 10.1042/cs20150660 26888560 PMC5513728

[B39] Tan X. Zhang X. Pan L. Tian X. Dong P. (2017). Identification of key pathways and genes in advanced coronary atherosclerosis using bioinformatics analysis. *Biomed. Res. Int.* 2017:4323496. 10.1155/2017/4323496 29226137 PMC5684517

[B40] Vignali D. A. Collison L. W. Workman C. J. (2008). How regulatory T cells work. *Nat. Rev. Immunol.* 8 523–532. 10.1038/nri2343 18566595 PMC2665249

[B41] Violi F. Carnevale R. Loffredo L. Pignatelli P. Gallin J. I. (2017). NADPH Oxidase-2 and atherothrombosis: insight from chronic granulomatous disease. *Arterioscler. Thromb. Vasc. Biol.* 37 218–225. 10.1161/atvbaha.116.308351 27932349

[B42] Wang C. H. Shi H. H. Chen L. H. Li X. L. Cao G. L. Hu X. F. (2019). Identification of Key lncRNAs associated with atherosclerosis progression based on public datasets. *Front. Genet.* 10:123. 10.3389/fgene.2019.00123 30873207 PMC6403132

[B43] Wang B. Chen L. Dai L. Fang W. Wang H. (2019). Alisol B 23-acetate ameliorates lipopolysaccharide-induced cardiac dysfunction by suppressing toll-like receptor 4 (TLR4)/NADPH Oxidase 2 (NOX2) signaling pathway. *Med. Sci. Monit.* 25 8472–8481. 10.12659/msm.918252 31707400 PMC6863037

[B44] Wang Y. Liu T. Liu Y. Chen J. Xin B. Wu M. (2019). Coronary artery disease associated specific modules and feature genes revealed by integrative methods of WGCNA. MetaDE and machine learning. *Gene* 710 122–130. 10.1016/j.gene.2019.05.010 31075415

[B45] Wang H. Gu J. Hou X. Chen J. Yang N. Liu Y. (2017). Anti-inflammatory effect of miltirone on inflammatory bowel disease via TLR4/NF-κB/IQGAP2 signaling pathway. *Biomed. Pharmacother.* 85 531–540. 10.1016/j.biopha.2016.11.061 27903427

[B46] Wang J. Wei B. Cao S. Xu F. Chen W. Lin H. (2014). Identification by microarray technology of key genes involved in the progression of carotid atherosclerotic plaque. *Genes Genet. Syst.* 89 253–258. 10.1266/ggs.89.253 25948119

[B47] Williams H. Cassorla G. Pertsoulis N. Patel V. Vicaretti M. Marmash N. (2017). Human classical monocytes display unbalanced M1/M2 phenotype with increased atherosclerotic risk and presence of disease. *Int. Angiol.* 36 145–155. 10.23736/s0392-9590.16.03661-0 26871397

[B48] Wolf D. Ley K. (2019). Immunity and inflammation in atherosclerosis. *Circ. Res.* 124 315–327. 10.1161/circresaha.118.313591 30653442 PMC6342482

[B49] Zhang B. Horvath S. (2005). A general framework for weighted gene co-expression network analysis. *Stat. Appl. Genet. Mol. Biol.* 4:Article17. 10.2202/1544-6115.1128 16646834

[B50] Zhang J. Nie Q. Si C. Wang C. Chen Y. Sun W. (2019). weighted gene co-expression network analysis for RNA-Sequencing data of the varicose veins transcriptome. *Front. Physiol.* 10:278. 10.3389/fphys.2019.00278 30941060 PMC6433941

[B51] Zhou S. Liu S. Liu X. Zhuang W. (2020). Bioinformatics gene analysis of potential biomarkers and therapeutic targets for unstable atherosclerotic plaque-related stroke. *J. Mol. Neurosci.* 10.1007/s12031-020-01725-2 33155176

[B52] Zirlik A. Lutgens E. (2015). An inflammatory link in atherosclerosis and obesity. Co-stimulatory molecules. *Hamostaseologie* 35 272–278. 10.5482/hamo-14-12-0079 26225729

