# Supplementary figures and images for: Weighted Gene Co-expression Network Analysis Identifies Crucial Genes Mediating Progression of Carotid Plaque
Source: Front Physiol. 2021 Feb 5;12:601952. doi: 10.3389/fphys.2021.601952 (PMC7894049; doi:10.3389/fphys.2021.601952)

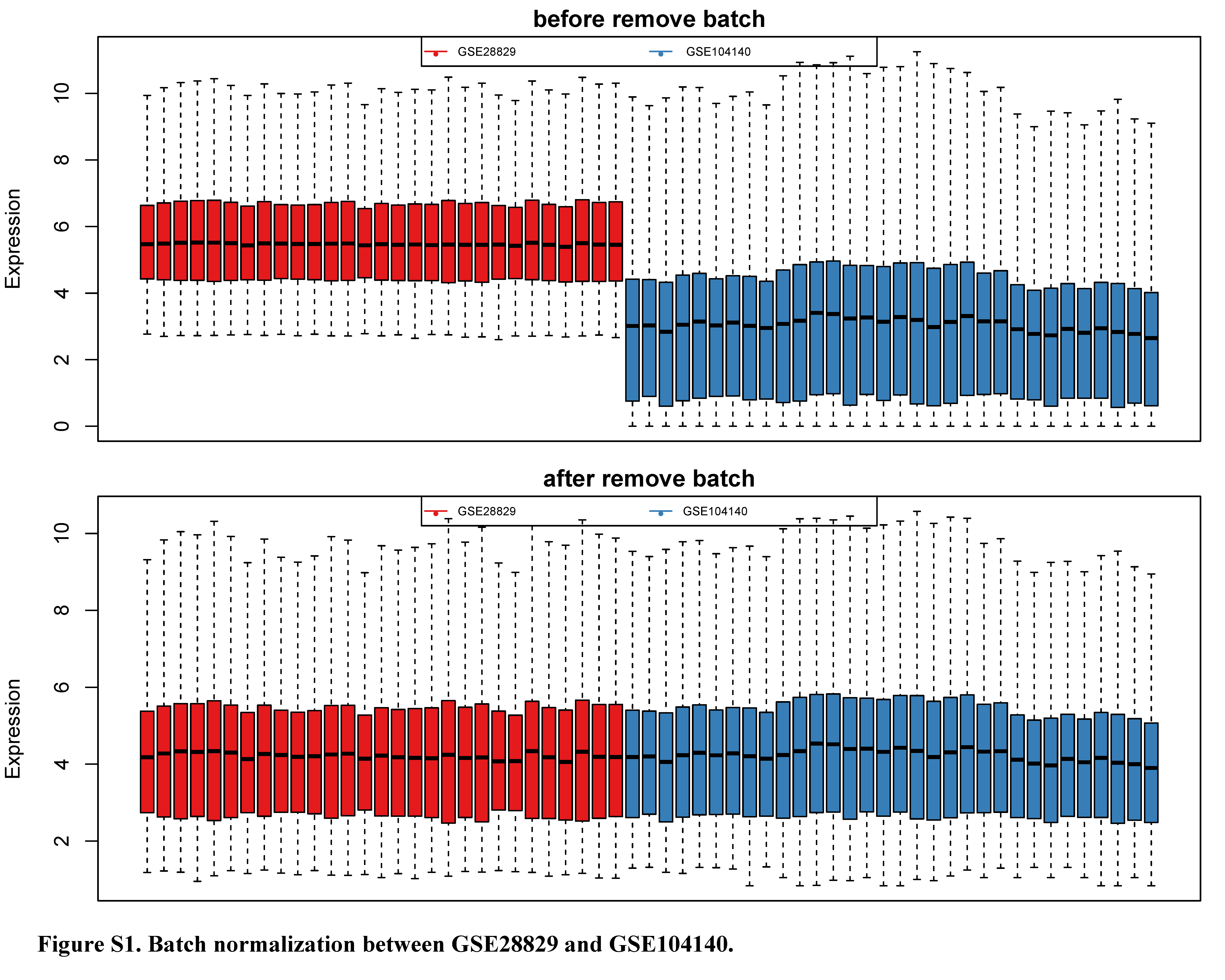

Supplement: Supplementary Figure 1 — Batch normalization between GSE28829 and GSE104140. [file Image_1.TIF]

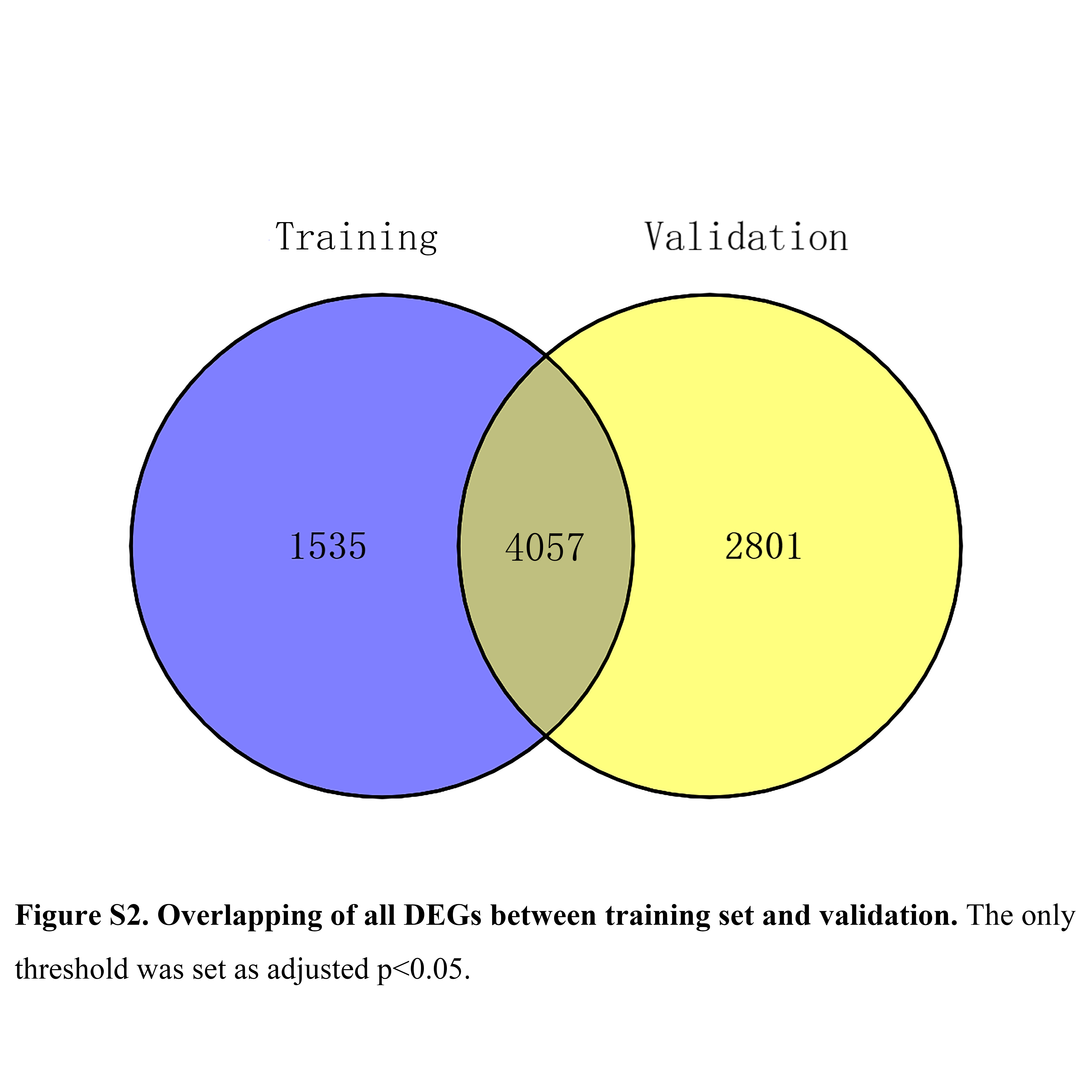

Supplement: Supplementary Figure 2 — Overlapping of all DEGs between training set and validation. The only threshold was set as adjusted p < 0.05. [file Image_2.TIF]

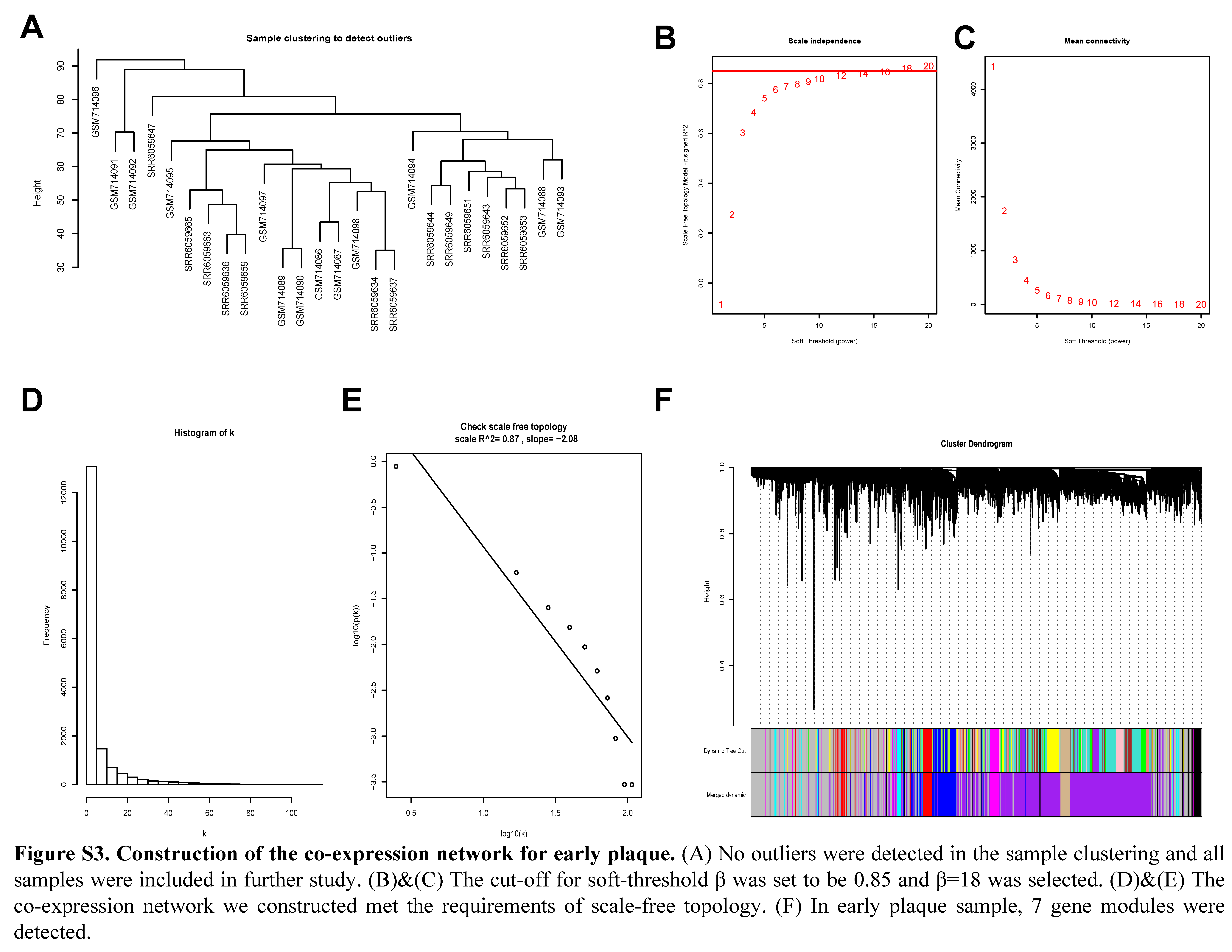

Supplement: Supplementary Figure 3 — Construction of the co-expression network for early plaque. (A) No outliers were detected in the sample clustering and all samples were included in further study. (B,C) The cut-off for soft-threshold β was set to be 0.85 and β = 18 was selected. (D,E) The co-expression network we constructed met the requirements of scale-free topology. (F) In early plaque sample, 7 gene modules were detected. [file Image_3.TIF]

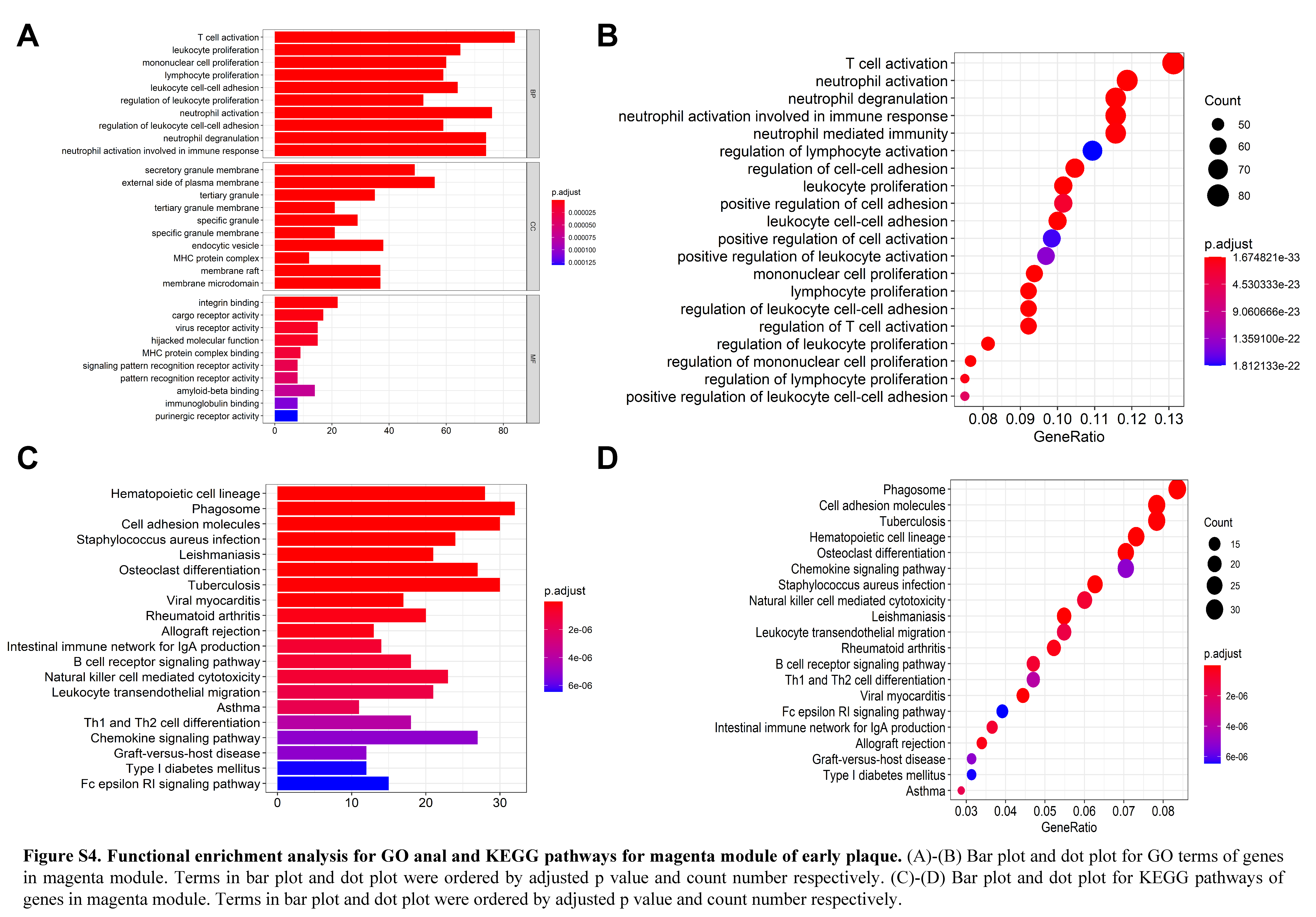

Supplement: Supplementary Figure 4 — Functional enrichment analysis for GO anal and KEGG pathways for magenta module of early plaque. (A,B) Bar plot and dot plot for GO terms of genes in magenta module. Terms in bar plot and dot plot were ordered by adjusted p value and count number respectively. (C,D) Bar plot and dot plot for KEGG pathways of genes in magenta module. Terms in bar plot and dot plot were ordered by adjusted p value and count number, respectively. [file Image_4.tif]

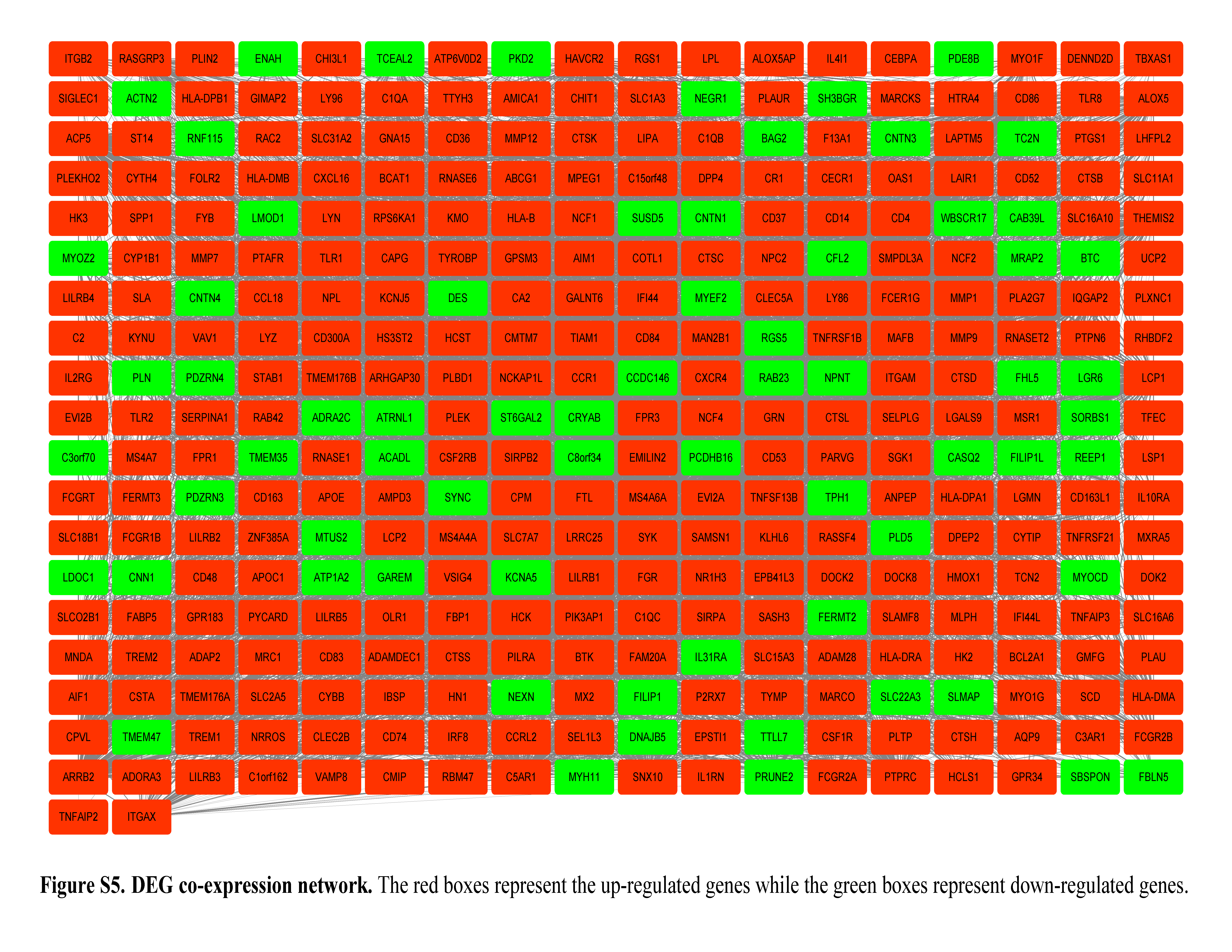

Supplement: Supplementary Figure 5 — DEG co-expressionnetwork. The red boxes represent the up-regulated genes while the green boxes represent down-regulated genes. [file Image_5.tif]

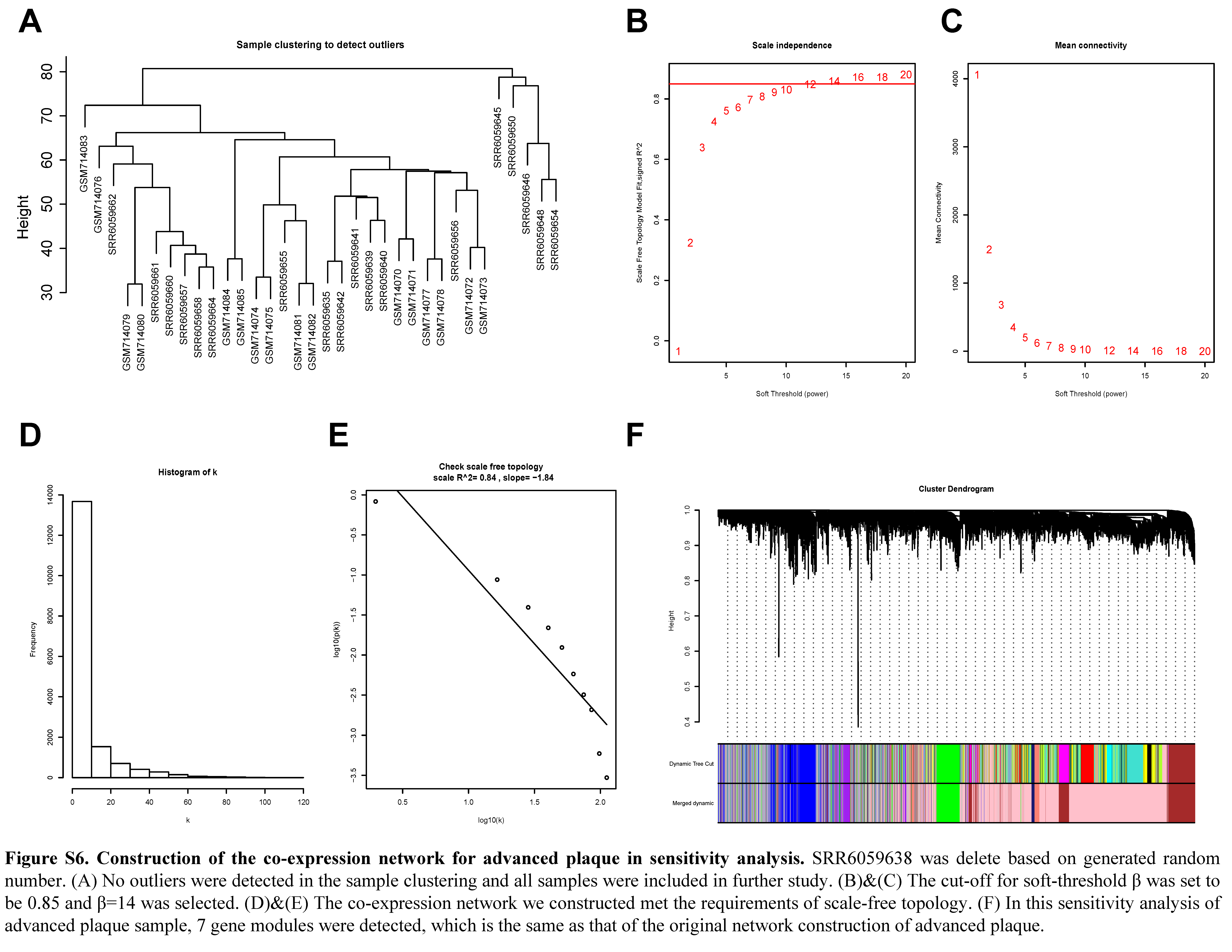

Supplement: Supplementary Figure 6 — Construction of the co-expression network for advanced plaque in sensitivity analysis. SRR6059638 was delete based on generated random number. (A) No outliers were detected in the sample clustering and all samples were included in further study. (B,C) The cut-off for soft-threshold β was set to be 0.85 and β = 14 was selected. (D,E) The co-expression network we constructed met the requirements of scale-free topology. (F) In this sensitivity analysis of advanced plaque sample, 7 gene modules were detected, which is the same as that of the original network construction of advanced plaque. [file Image_6.TIF]

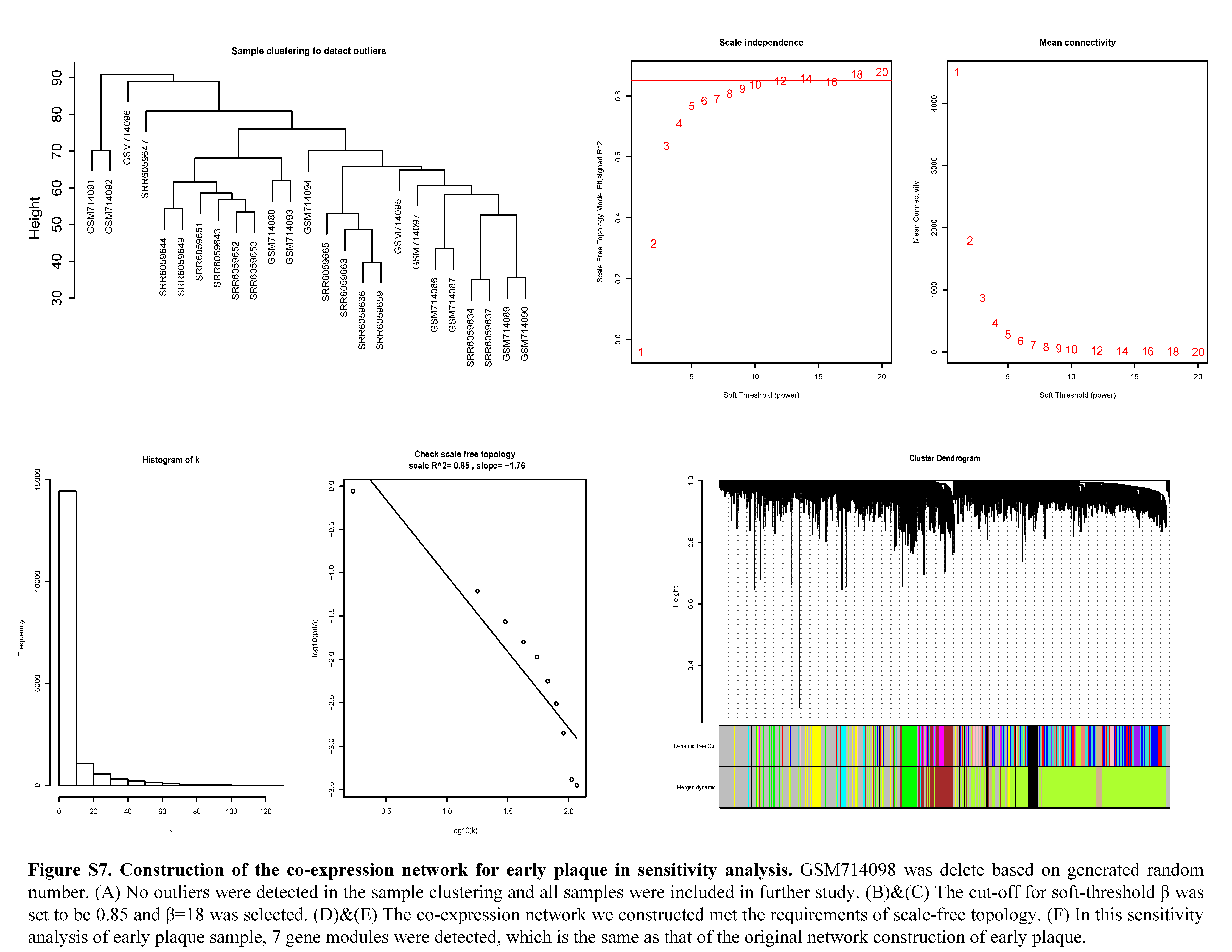

Supplement: Supplementary Figure 7 — Construction of the co-expression network for early plaque in sensitivity analysis. GSM714098 was delete based on generated random number. (A) No outliers were detected in the sample clustering and all samples were included in further study. (B,C) The cut-off for soft-threshold β was set to be 0.85 and β = 18 was selected. (D,E) The co-expression network we constructed met the requirements of scale-free topology. (F) In this sensitivity analysis of early plaque sample, 7 gene modules were detected, which is the same as that of the original network construction of early plaque. [file Image_7.TIF]

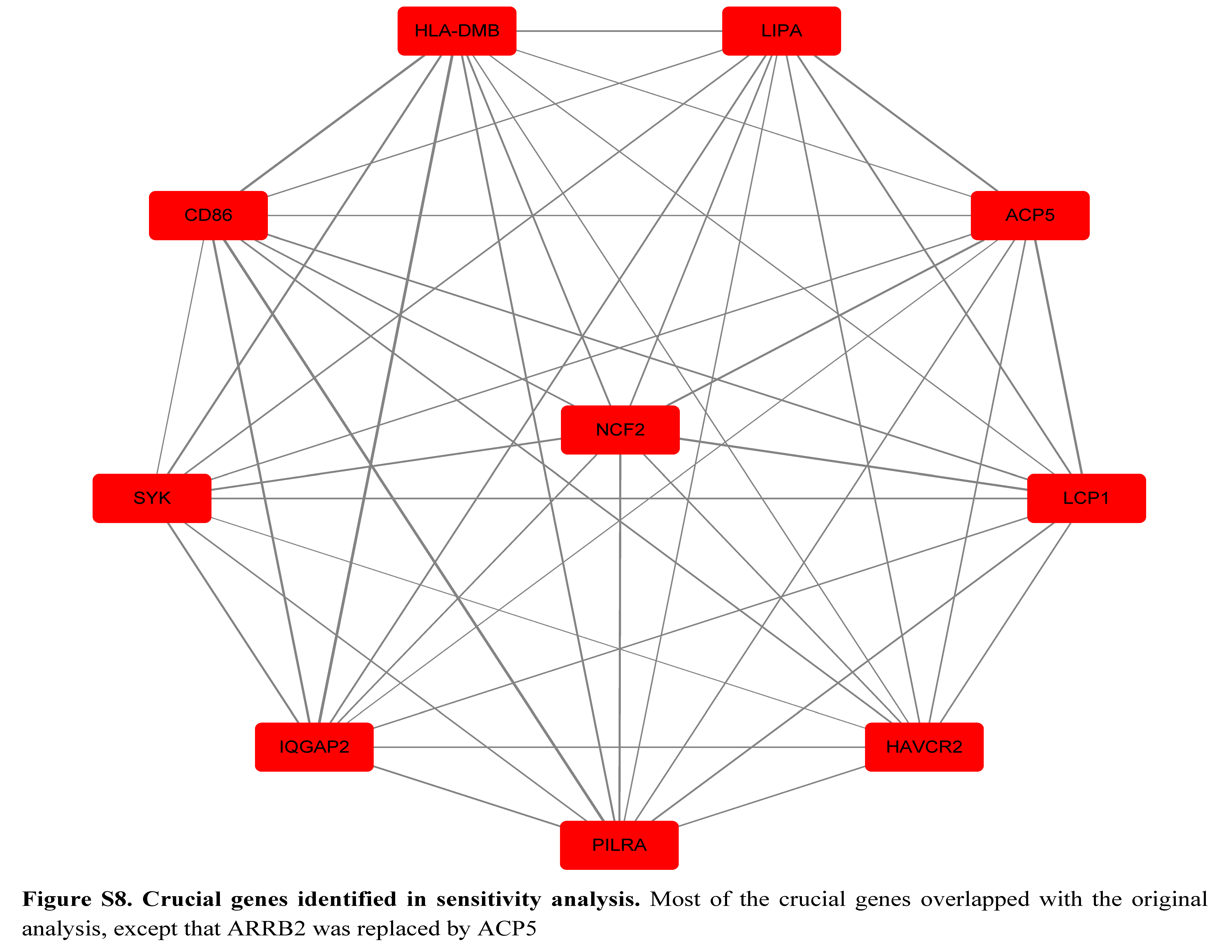

Supplement: Supplementary Figure 8 — Crucial genes identified in sensitivity analysis. Most of the crucial genes overlapped with the original analysis, except that ARRB2 was replaced by ACP5 [file Image_8.TIF]
