# Supplementary material for: Weighted Gene Co-expression Network Analysis Identifies Crucial Genes Mediating Progression of Carotid Plaque
Source: Front Physiol. 2021 Feb 5;12:601952. doi: 10.3389/fphys.2021.601952 (PMC7894049; doi:10.3389/fphys.2021.601952)
Supplement: Supplementary Table 5 — Hub genes for each module of early plaque and advanced plaque. [file Table_5.DOCX]

**Table S5. Hub genes for each module of early plaque and advanced plaque**

| **Module** | **Gene symbol** | **Official full name** | **MM** | |  |
| --- | --- | --- | --- | --- | --- |
| *Early plaque* |  |  |  | |  |
| black | *RAP1GAP* | RAP1 GTPase activating protein | 0.924181992 | |  |
| blue | *CCDC185* | coiled-coil domain containing 185 | 0.938742581 | |  |
| cyan | *STMN2* | stathmin 2 | | 0.944621884 | |
| magenta | *FPR3* | formyl peptide receptor 3 | 0.892491397 | |  |
| purple | *USP8* | ubiquitin specific peptidase 8 | 0.952999887 | |  |
| red | *ALPP* | alkaline phosphatase, placental | 0.905320234 | |  |
| tan | *C7orf50* | chromosome 7 open reading frame 50 | 0.964149940 | |  |
| *Advanced plaque* |  |  |  | |  |
| black | *ARHGAP18* | Rho GTPase activating protein 18 | 0.917831092 | |  |
| blue | *PCDH7* | protocadherin 7 | 0.928301543 | |  |
| greenyellow | *PRPF4B* | pre-mRNA processing factor 4B | 0.940750498 | |  |
| midnightblue | *RPL27* | ribosomal protein L27 | 0.92694162 | |  |
| pink | *TTC6* | tetratricopeptide repeat domain 6 | 0.914865114 | |  |
| salmon | *EMILIN1* | elastin microfibril interfacer 1 | 0.900543484 | |  |
| yellow | *VCX2* | variable charge X-linked 2 | 0.924904978 | |  |
