# Supplementary material for: Weighted Gene Co-expression Network Analysis Identifies Crucial Genes Mediating Progression of Carotid Plaque
Source: Front Physiol. 2021 Feb 5;12:601952. doi: 10.3389/fphys.2021.601952 (PMC7894049; doi:10.3389/fphys.2021.601952)
Supplement: Supplementary Table 7 — Coefficients of lasso regression. [file Table_7.DOCX]

**Table S7. Coefficients of lasso regression**

| **Gene** | **Coefficients** |
| --- | --- |
| IQGAP2 | -0.16700816 |
| FPR3 | -0.098814167 |
| FCER1G | -0.068749729 |
| SLC1A3 | -0.01252224 |
| C5AR1 | -0.008505258 |
| PLA2G7 | -0.007269865 |
| ALOX5 | 0.01110583 |
| CCR1 | 0.021218576 |
| RASGRP3 | 0.066041667 |
| SLAMF8 | 0.084730922 |
| C3AR1 | 0.086439442 |
| AIF1 | 0.102591065 |
| AMPD3 | 0.145539238 |
| BTK | 0.146746152 |
| CTSB | 0.193964174 |
